# Empyema and pyogenic spondylitis caused by direct *Streptococcus gordonii* infection after a compression fracture: a case report

**DOI:** 10.1186/s40792-019-0613-x

**Published:** 2019-04-03

**Authors:** Daisuke Nakamura, Ryoichi Kondo, Akiko Makiuchi, Kenichi Isobe

**Affiliations:** 1 Department of Thoracic Surgery, National Hospital Organization Matsumoto Medical Center, 2-20-30 Murai-Machi-Minami, Matsumoto, 399-0021 Japan; 2 Department of Orthopedics, National Hospital Organization Matsumoto Medical Center, 2-20-30 Murai-Machi-Minami, Matsumoto, 399-0021 Japan

**Keywords:** Empyema, Compression fracture, Video-assisted thoracic surgery, Pyogenic spondylitis

## Abstract

**Background:**

Empyema and pyogenic spondylitis are common diseases that are often caused by oral pathogens in direct or hematogenous infection. However, there exists no report describing empyema and pyogenic spondylitis caused by oral pathogens after a compression fracture of the vertebral body. Herein, we report a case of empyema and pyogenic spondylitis caused by direct *Streptococcus gordonii* infection after a compression fracture of the vertebral body.

**Case presentation:**

A 74-year-old man had back pain while working. At 1 week after experiencing back pain, he underwent periodontal debridement. At 3 weeks after periodontal debridement, he visited our hospital owing to the absence of improvement in back pain. He was admitted on the same day with a diagnosis of compression fracture of the 12th thoracic vertebral body. Magnetic resonance imaging (MRI) revealed a compression fracture of the 12th thoracic vertebral body and a hematoma anterior to the vertebral body. Computed tomography (CT) showed no findings suggestive of infection. After admission, antibiotic therapy was initiated, as the patient developed fever and his blood cultures grew *S. gordonii*. CT performed after antibiotic therapy revealed a right-sided pleural effusion, and drainage was performed. As the inflammation did not improve after thoracic drainage for empyema, surgical debridement through video-assisted thoracic surgery was performed. Intraoperative pleural effusion cultures also grew *S. gordonii*. Postoperative MRI showed low T1-weighted signals and high T2-weighted signals in the 12th thoracic vertebral body, and the signals spread to the upper and lower intervertebral disk space; hence, a diagnosis of empyema and pyogenic spondylitis due to direct infection spread was established. Intravenous antibiotic therapy was continued for 6 weeks and then was switched to oral antibiotic treatment. His C-reactive protein level and erythrocyte sedimentation rate gradually decreased and remained within normal limits. Neither empyema nor pyogenic spondylitis had recurred at 12 months after surgery.

**Conclusions:**

Compression fracture with dental procedures possibly results in the thoracic cavity and spinal infection caused by oral pathogens. We emphasize the importance of early imaging examinations, diagnosis, and appropriate treatment for patients with compression fractures who develop a fever.

## Background

Empyema and pyogenic spondylitis are common diseases, and their early diagnosis and appropriate treatment can shorten hospital stay and reduce mortality. Both empyema and pyogenic spondylitis are often caused by oral pathogens in direct or hematogenous infection. However, there exists no report describing empyema and pyogenic spondylitis caused by oral pathogens after a compression fracture of the vertebral body. Herein, we report a case of empyema and pyogenic spondylitis caused by direct *Streptococcus gordonii* infection after a compression fracture of the vertebral body.

## Case presentation

**Fig. 1:**
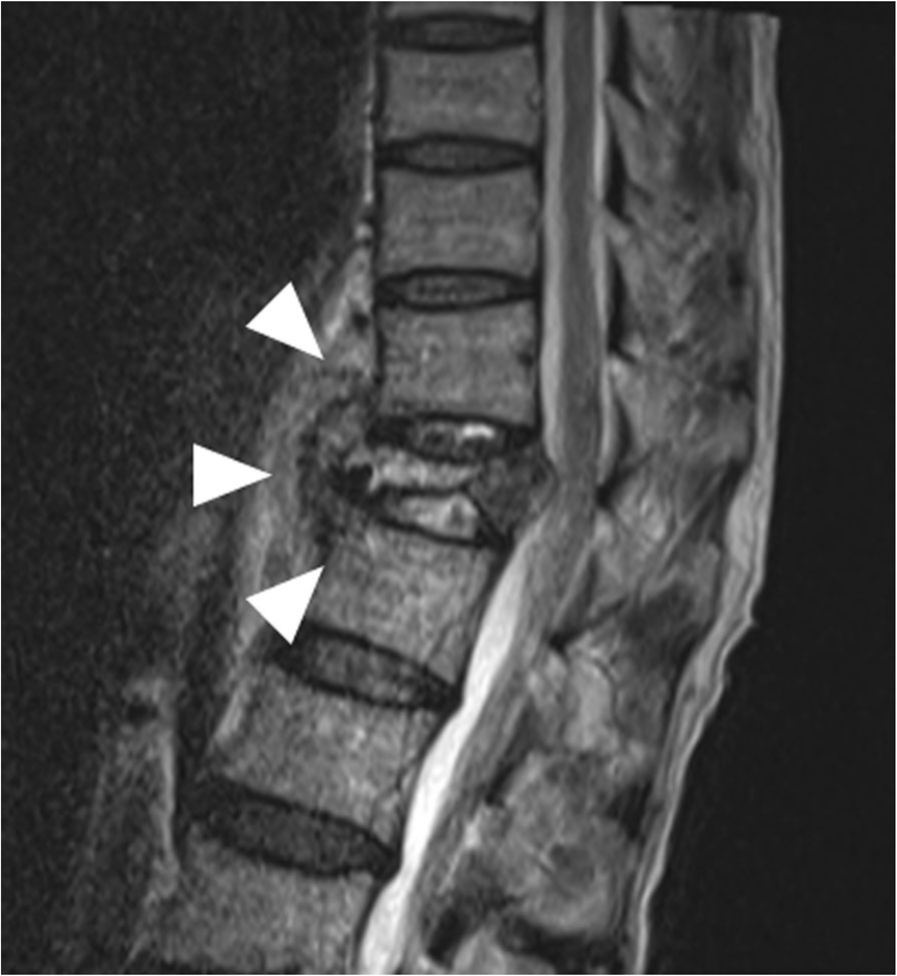
Lumbar magnetic resonance imaging (MRI) on admission. MRI revealed a compression fracture of the 12th thoracic vertebral body and hematoma anterior to the vertebral body (white arrowheads)

**Fig. 2:**
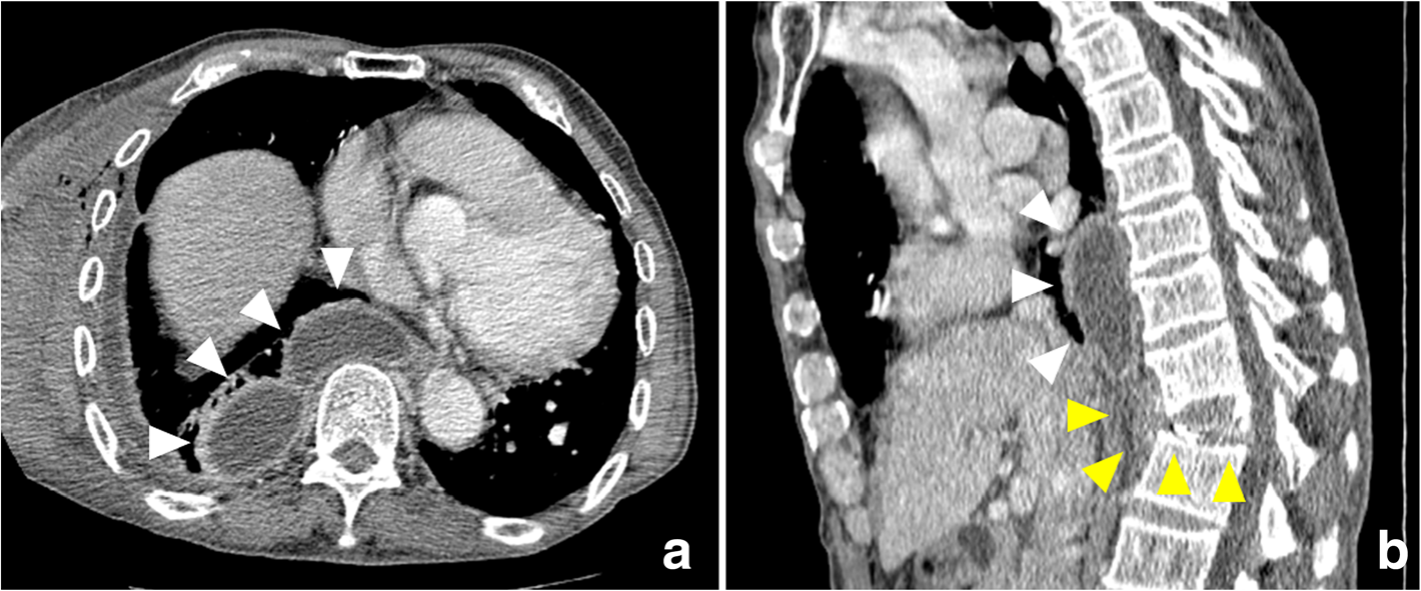
Preoperative enhanced chest computed tomography (CT). Enhanced chest CT revealed a right-sided encapsulated pleural effusion (white arrowheads) and hematoma anterior to the compression fracture of the vertebral body (yellow arrowheads). **a** Axial view. **b** Sagittal view

**Fig. 3:**
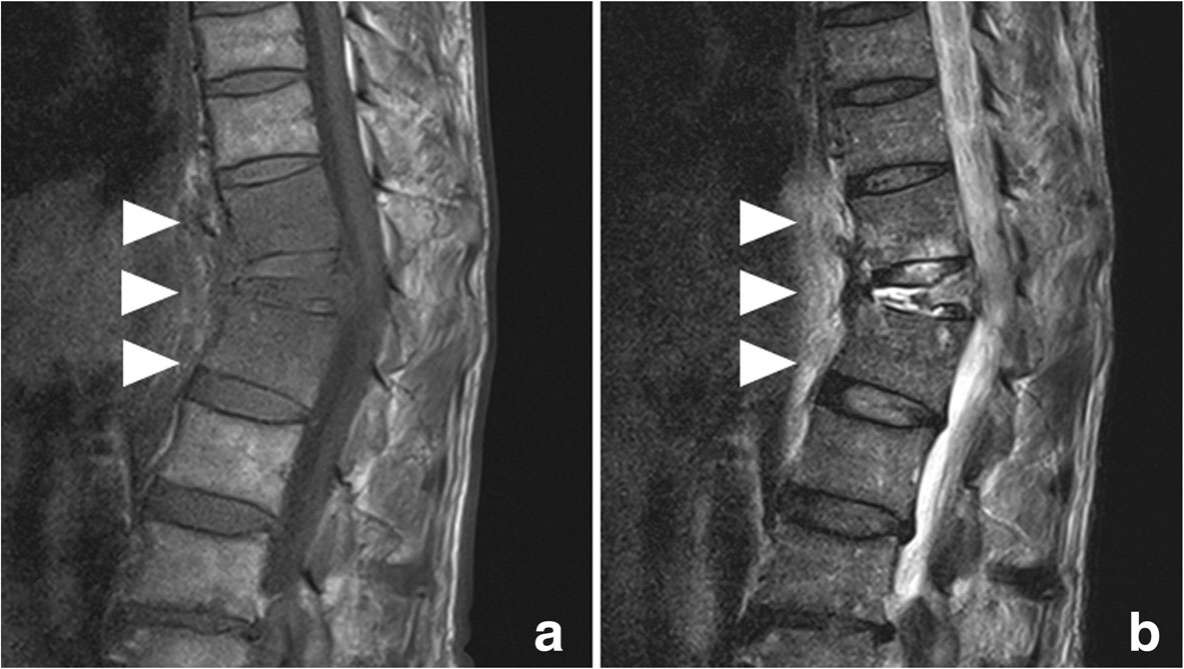
Postoperative lumbar MRI. **a**, **b** MRI revealed low T1-weighted signals and high T2-weighted signals in the 12th thoracic vertebral body, and the signals spread to the upper and lower intervertebral disk space (white arrowheads)

A 74-year-old man had back pain while working. At 1 week after experiencing back pain, he underwent periodontal debridement. He did not take antibiotics despite their prescription and developed a fever after debridement. At 3 weeks after periodontal debridement, he visited our hospital because back pain had not improved. He was admitted on the same day in the Department of Orthopedics with a diagnosis of compression fracture of the 12th thoracic vertebral body. He had never smoked and had no history of diabetes mellitus or steroid therapy. Physical examination revealed a temperature of 38.5 °C and severe lumbar back pain. His laboratory test results indicated a peripheral white blood cell (WBC) count of 8760/μL, an erythrocyte sedimentation rate (ESR) of 75 mm/h, and a C-reactive protein (CRP) level of 9.9 mg/dL. Magnetic resonance imaging (MRI) revealed a compression fracture of the 12th thoracic vertebral body and a hematoma anterior to the vertebral body (Fig. 1). Chest and abdominal computed tomography (CT) showed no findings suggestive of infection and hematoma anterior to the vertebral body. Orthopedic surgeons initially managed the compression fracture using a corset. Furthermore, we performed a percutaneous biopsy of the vertebral body at 15 days after admission because the patient’s fever did not resolve. Cytological findings revealed no malignancy, and biopsy cultures were negative. During follow-up observation without antibiotic therapy, blood culture was performed at 20 days after admission because his inflammation worsened (peripheral WBC count, 9490/μL; CRP level, 22.4 mg/dL). Two blood cultures grew *S. gordonii* susceptible to cefaclor (minimum inhibitory concentration [MIC], 0.5), cefotaxime (MIC, 0.12), erythromycin (MIC, 0.12), meropenem (MIC, 0.12), clindamycin (MIC, 0.25), and vancomycin (MIC, 1). Cardiac ultrasonography showed no infective endocarditis. Immediately after the detection of *S. gordonii* in blood cultures, we initiated empirical antibiotic therapy with intravenous (IV) meropenem (1.5 g/day) at 26 days after admission. As the patient verbalized an increase in back pain, CT was performed at 5 days after initial antibiotic therapy, which showed right-sided pleural effusion encapsulated in the right thoracic cavity (Fig. 2a, b). Thoracic surgeons were consulted about pleural effusion, and we placed a 20-Fr thoracic drain at the dorsal side of the right thoracic cavity from the right fifth intercostal space. We established a diagnosis of empyema because the effluent from the drain was purulent and the pleural effusion culture yielded *S. gordonii*. The inflammation did not improve (peripheral WBC count, 12,320/μL; CRP level, 20.0 mg/dL), and pleural effusion encapsulated in the thoracic cavity was still observed on chest CT at 4 days after drainage; hence, we performed surgical debridement through video-assisted thoracic surgery (VATS). The lungs had extensively adhered to the chest wall. When we peeled the adhesion, a large amount of purulent pleural effusion was observed in the space anterior to the vertebral body and was drained as much as possible. Adhesion was severe at the dorsal side of the thoracic cavity, particularly at the anterior aspect of the vertebral body. No evidence of apparent vertebral body fracture or damage to the parietal pleura was observed during surgery. After drainage, one drain was placed in the space anterior to the vertebral body and another drain in the lung apex, and the operation was completed. Intraoperative pleural effusion cultures also yielded *S. gordonii*. A drainage tube was displaced at 4 days after surgery. Because of the patient’s mild but persistent back pain, MRI was performed at 10 days after surgery to evaluate the compression fracture. MRI showed low T1-weighted signals and high T2-weighted signals in the 12th thoracic vertebral body, and the signals spread to the upper and lower intervertebral disk space (Fig. 3a, b). We diagnosed bacteremia and an infected hematoma resulting from periodontal debridement after a compression fracture; the infected site directly spread and caused empyema and pyogenic spondylitis. The patient postoperatively received IV antibiotic therapy with meropenem (1.5 g/day) and clindamycin (1200 mg/day) for 2 weeks and with ceftriaxone (3.0 g/day) and clindamycin (1200 mg/day) for another 4 weeks. A dentist was consulted for dental caries assessment after surgery and provided continuous treatment for dental caries during hospitalization. Follow-up CT after treatment revealed resolution of pleural effusion, and the patient’s back pain gradually decreased after surgery. After discharge, oral treatment with clindamycin (600 mg/day) and minocycline (200 mg/day) was maintained for 5 weeks. Because of a taste disorder and reduced renal function due to minocycline, the oral treatment was changed from clindamycin and minocycline to cefcapene (300 mg/day). Treatment with cefcapene was continued for 9 weeks and completed. CRP level and ESR gradually decreased and remained within normal limits. Neither empyema nor pyogenic spondylitis had recurred at 12 months after surgery.

## Discussion

We reported a case of empyema and pyogenic spondylitis caused by direct *S. gordonii* infection after a compression fracture of the vertebral body. To our knowledge, this is the first report describing empyema and pyogenic spondylitis due to oral pathogen infection after a compression fracture.

Empyema is a common disease resulting from pneumonia, postoperative complications, and trauma, particularly in elderly individuals, diabetic patients, and immunocompromised patients. Early diagnosis and complete drainage through VATS can shorten the hospital stay and reduce mortality. Empyema is relatively frequently caused by oral pathogens; however, empyema caused by *S. gordonii* is extremely rare, with only one case being reported in the literature [1]. *S. gordonii* is a Gram-positive, non-motile, coccus belonging to the viridians streptococcal group [2]. Generally present in the mouth and upper airway, this facultative anaerobe is capable of spreading to extraoral sites and causing infective endocarditis, septic arthritis, and toxic shock-like syndrome [3, 4]. Furthermore, some reports have described empyema caused by periodontal disease [5]. A route for dental infection to spread to the mediastinum and thoracic cavity via the neck and a route for bacteria to reach the thoracic cavity via blood are possible.

Pyogenic spondylitis is also usually due to hematogenous infection via the arterial and venous routes and direct infection spread. Essential elements for the successful treatment of pyogenic spondylitis include fixation of the affected section of the spinal column and antibiotic therapy. Pyogenic spondylitis after a compression fracture of the vertebral body is rare, with only 14 cases reported in the literature [6]. Further, there is no report on empyema after a compression fracture of the vertebral body. In order for pyogenic spondylitis to develop after a compression fracture, the coexistence of both fracture and bacteremia is required. Some areas of blood stagnation have been observed with vertebral fracture under these conditions. Once blood stagnation occurs, bacterial inoculation could arise through the end-arterial arcades, resulting in pyogenic spondylitis [7]. Circulation to the endplates of the collapsed vertebral bodies is impaired by the bone fragments, and the same site becomes easily infected with further formation of hematoma [8].

**Fig. 4:**
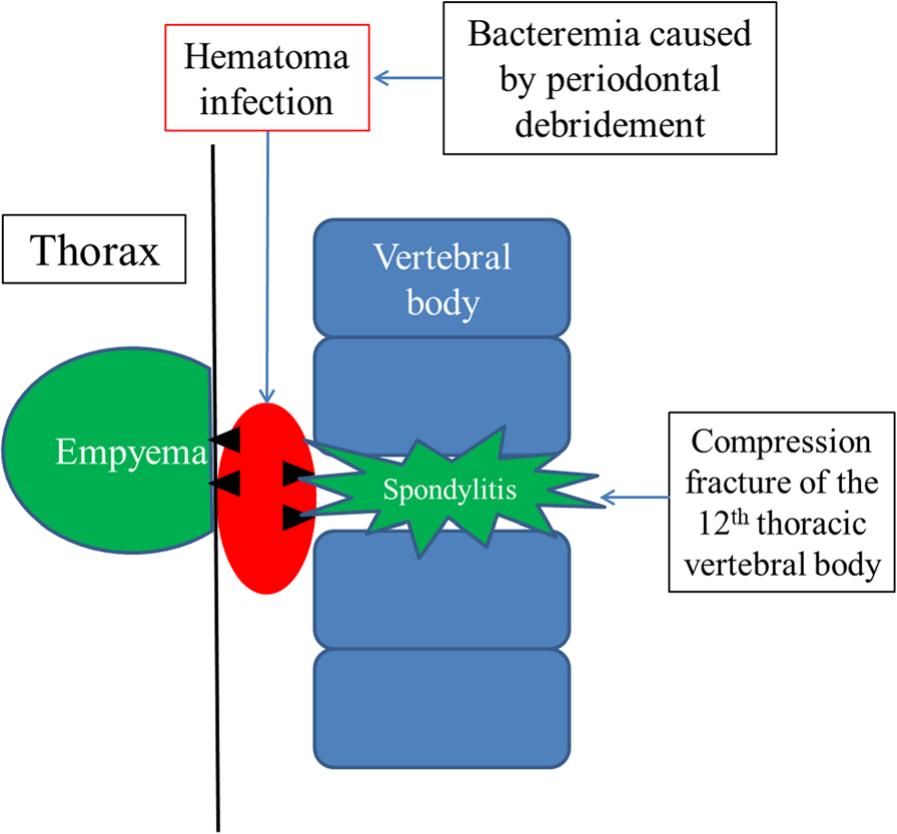
Schema shows the mechanism by which infected hematoma caused empyema and pyogenic spondylitis

Temporary bacteremia often develops after tooth extraction or periodontal debridement at a rate of 60–80%, as confirmed by blood cultures performed immediately after dental treatment [9]. Oral bacteria have been well reported to hematogenously spread after dental procedures, leading to spinal infection [10]. In our case, the patient underwent periodontal debridement after a compression fracture of the vertebral body. Similar to the mechanism of pyogenic spondylitis formation, the compression fracture resulted in an easily infected site. In addition, periodontal debridement led to bacteremia and infected hematoma. It is thought that this infected lesion directly spreads into the thoracic cavity and vertebral body, resulting in empyema and pyogenic spondylitis (Fig. 4). Because the vertebral body may show high T2-weighted signals on MRI even in the case of a typical compression fracture and the upper and lower intervertebral discs of the vertebral bodies with compression fracture were not examined for damage using lumbar MRI on admission, we initially did not consider infection. In addition to compression fractures, hematologic diseases such as malignant lymphoma and myeloma were considered as causes of fever and CRP level elevation. Various blood tests and biopsy for differential diagnosis were performed after hospitalization; nonetheless, no significant blood test or cytological findings were observed. Even if it was an infectious disease, the target bacteria were unknown in biopsy cultures; hence, follow-up observation was continued without antibiotic therapy. Consequently, the non-provision of antibiotic therapy despite persistent fever during hospitalization was one of the reasons for the spread of infection; hence, antibiotic therapy should be initiated as soon as possible. We diagnosed empyema and pyogenic spondylitis, performed surgical debridement through VATS, and provided antibiotic therapy as soon as possible. With these treatments, inflammation could be reduced, and a good course was achieved.

## Conclusions

In conclusion, the lack of typical imaging findings led to a delay in diagnosis. Compression fracture with dental procedures possibly results in the thoracic cavity and spinal infection caused by oral pathogens. We emphasize the importance of early imaging examinations, diagnosis, and appropriate treatment for cases in which patients with compression fracture develop a fever.

## Abbreviations

CRP: C-reactive protein
CT: Computed tomography
ESR: Erythrocyte sedimentation rate
IV: Intravenous
MIC: Minimum inhibitory concentration
MRI: Magnetic resonance imaging
VATS: Video-assisted thoracic surgery
WBC: White blood cell

## References

[1] Krantz AM, Ratnaraj F, Velagapudi M, Krishnan M, Gujjula NR, Foral PA (2017). Streptococcus gordonii empyema: a case report and review of empyema. Cureus..

[2] Kilian M, Mikkelsen L, Henrichsen J (1989). Taxonomic study of viridans streptococci: description of Streptococcus gordonii sp. nov. and emended descriptions of Streptococcus sanguis (White and Niven 1946), Streptococcus oralis (Bridge and Sneath 1982), and Streptococcus mitis (Andrewes and Horder 1906). Int J Syst Evol.

[3] Yombi Jc BL, Jonckheere S, Wilmes D, Cornu O, Vandercam B (2012). Streptococcus gordonii septic arthritis: two cases and review of literature. BMC Infect Dis.

[4] Liao CY, Su KJ, Lin CH, Huang SF, Chin HK, Chang CW (2016). Plantar purpura as the initial presentation of viridians streptococcal shock syndrome secondary to Streptococcus gordonii bacteremia. Can J Infect Dis Med Microbiol.

[5] Rallis G, Papadakis D, Koumoura F, Gakidis I, Mihos P (2006). Rare complications of a dental abscess. Gen Dent.

[6] Imabayashi H, Hosogane N, Matsuhashi Y, Yato Y, Asazuma T, Nemoto K (2015). Pyogenic spondylitis initially presenting vertebral compression fracture: report of three cases. J Spine Res.

[7] Sapico FL, Montgomerie JZ (1990). Vertebral osteomyelitis. Infect Dis Clin N Am.

[8] McHenry MC, Duchesneau PM, Keys TF, Rehm SJ, Boumphrey FR (1988). Vertebral osteomyelitis presenting as spinal compression fracture: six patients with underlying osteoporosis. Arch Intern Med.

[9] Tomás I, Alvarez M, Limeres J, Potel C, Medina J, Diz P (2007). Prevalence, duration and aetiology of bacteraemia following dental extractions. Oral Dis.

[10] Muzii VF, Mariottini A, Zalaffi A, Carangelo BR, Palma L (2006). Cervical spine epidural abscess: experience with microsurgical treatment in eight cases. J Neurosurg Spine.

